# The Role of Host‐Range Expansion and Co‐Speciation in Host–Parasite Associations With the Divergence of the Great Tit Species Complex

**DOI:** 10.1002/ece3.70859

**Published:** 2025-01-21

**Authors:** Xi Huang, Vincenzo A. Ellis, Yangyang Peng, Farah Ishtiaq, Haitao Wang, Wei Liang, Qiang Wu, Staffan Bensch, Lu Dong

**Affiliations:** ^1^ MOE Key Laboratory for Biodiversity Science and Ecological Engineering, College of Life Sciences Beijing Normal University Beijing China; ^2^ Deptartment of Entomology and Wildlife Ecology University of Delaware Newark Delaware USA; ^3^ Centre in InStem Tata Institute for Genetics and Society Bangalore India; ^4^ Jilin Engineering Laboratory for Avian Ecology and Conservation Genetics, School of Life Sciences Northeast Normal University Changchun China; ^5^ Ministry of Education Key Laboratory for Ecology of Tropical Islands, College of Life Sciences Hainan Normal University Haikou China; ^6^ MOE Key Laboratory of Biosystems Homeostasis & Protection, College of Life Sciences Zhejiang University Hangzhou China; ^7^ Department of Biology Lund University Lund Sweden

**Keywords:** haemosporidian, host–parasite association, host‐range expansion, *Parus major*, phylogeography

## Abstract

During the evolution of parasites, co‐speciation and host‐range expansion are thought to play roles in establishing associations with hosts, while sorting events can lead to dissolution of those associations. To address the roles of these processes, we focus on avian haemosporidian parasites infecting hosts of the intensively studied great tit species complex. We estimated the phylogeography of lineages detected in the species complex, and quantified their transition probabilities among hosts. Lineages detected in different host species presented a strong geographical signal but did not form monophyletic groups. Yet, distributions of lineages are not merely the result of their dispersal limitations, as many lineages that infect only one focal species can be found in birds sympatric with other focal species. Besides, closely related lineages that infect the same host species reach more similar rates of infection than expected by chance. Finally, *Haemoproteus* and *Leucocytozoon* lineages infecting *P. major* , the most recently dispersed species, were more generalized than others, consistent with a pattern of generalist parasites expanding their host ranges by infecting newly encountered host species. Our results suggest that host–parasite associations in this system are mainly the result of sorting events and host‐range expansion of parasites, rather than co‐speciation.

## Graphical abstract

In this work, we investigated the global phylogeographic pattern of host–parasite associations in a well‐studied common bird species complex, and the role of different ecological processes in establishing the patterns. We found that the parasite distributions are not merely the result of geographical restriction, but mainly shaped by sorting events and host range expansion during the host dispersal. It appears that co‐speciation, which is frequently overestimated, is less important in the establishment of host–parasite associations.

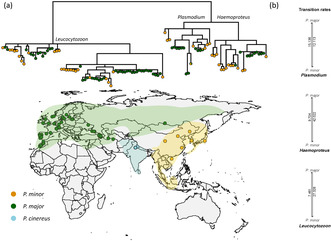

## 1 Introduction

**FIGURE 1:**
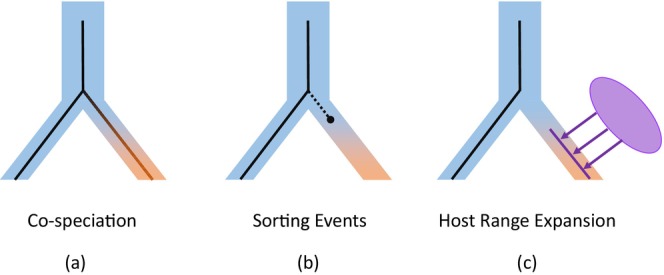
Scenarios of the establishment and dissolution of host–parasite associations: (a) co‐speciation, (b) sorting events, including “missing the boat” and parasite extinction, and (c) host shifts. Host species and their phylogenetic relationships are shaded and parasites and their phylogenetic relationships are inset with thin solid lines.

Understanding the evolutionary and ecological factors that shape host–parasite associations is fundamental to research on epidemiology. Furthermore, the significance of this topic has been repeatedly underlined by outbreaks of emerging infectious disease in recent decades (Jones et al. 2008). Novel host–parasite associations can be formed when a parasite expands its host range to a newly encountered host or co‐speciates with its current host (Doña et al. 2017; Nylin et al. 2018), and are dissolved through sorting events including several distinct occasions (Figure 1). For example, when uninfected hosts disperse to new areas where their parasites are not distributed, this is a type of sorting referred to as “missing the boat” (Clayton and Moore 1997). In another case, a parasite may disperse with its host but fail to be transmitted in the new areas, and therefore go extinct (Johnson et al. 2003).

Co‐speciation occurs when an ancestral host population harboring a certain parasite divides into two or more subpopulations, and both the host and the newly formed parasite subpopulations evolve into distinct species. Alternatively, the parasite may keep the ability to infect more than one of the host subpopulations if there is continuous gene flow among parasite populations, leading to an expanded host range of the parasite (Brant and Orti 2003). Co‐speciation can be identified by congruent host and parasite phylogenies. However, host and parasite phylogenies are frequently incongruent (Brooks 1979), suggesting that host‐range expansion may play a major role in parasite diversification (de Vienne et al. 2013; Alcala et al. 2017), especially for vector transmitted parasites (Ricklefs et al. 2014; Ciloglu et al. 2020). Two basic conditions must be met for a parasite to colonize a novel host and thereby initiate host‐range expansion and subsequently host shifts (Combes 2001). First, the parasite must have the opportunity to encounter the host. Second, the parasite and the host must be compatible, that is, the parasite can complete its life cycle within the host and be transmitted. Thus, a host range expansion occurs when compatible hosts and parasites newly encounter one another (Kuris et al. 2007). In many cases, a parasite expands to a new host which is phylogenetically closely related (Davies and Pedersen 2008) or ecologically similar (Clark and Clegg 2017) to the current host, although host shifts among unrelated species are also detected, and sympatric speciation may occur afterwards if there was no gene flow between parasites in different hosts (Ricklefs et al. 2014). Since a parasite is unlikely to encounter all of its compatible hosts, dispersal ability which restricted novel encounter opportunities is an important barrier to host range expansion (Clayton, Bush, and Johnson 2004).

Avian haemosporidians have long been used as a model system for understanding the evolutionary history and ecological pattern of host–parasite associations (Rivero and Gandon 2018; Fecchio et al. 2021; Huang 2021; Darío Hernandes Córdoba et al. 2024). Avian haemosporidians, including avian malaria (*Plasmodium*) and related parasites (*Haemoproteus* and *Leucocytozoon*), are a diverse group of protozoan parasites transmitted by blood‐sucking dipteran insects to birds all over the world (Bensch, Hellgren, and Pérez‐Tris 2009). Microscopy‐based investigations have identified over 250 morphologically described species, and molecular analyses have identified more than 5000 distinct genetic lineages based on the barcoding segment of the parasite mitochondrial cytochrome b (*cyt b*) gene (Bensch, Hellgren, and Pérez‐Tris 2009). Previous studies have shown evidence of host‐range expansion (Ricklefs, Fallon, and Bermingham 2004; Galen and Witt 2014; Ricklefs et al. 2014; Fecchio et al. 2019; Ciloglu et al. 2020) and localized adaptation (Ellis et al. 2015) in these parasites. However, few studies have examined parasite associations with a widely distributed host species or species complex, with sampling across the entire distribution of the species (but see Galen and Witt 2014).

The great tit ( *Parus major* ) species complex is a group of closely related, small passerine birds with a broad geographical distribution covering almost all of Eurasia and part of Africa, and rank among the most intensively studied bird species with a relatively clear evolutionary history (Song et al. 2020). Moreover, with 66 distinct lineages recorded in the global database of avian haemosporidian infections, MalAvi database (v.2.4.1, http://130.235.244.92/Malavi/, Bensch, Hellgren, and Pérez‐Tris 2009) is among the top five species that harbors the largest number of haemosporidian lineages, making it an ideal model to study the establishment of host–parasite associations across space and time. There are multiple opinions on species definitions within this species complex. Here we use the latest IOC World Bird List (Gill, Donsker, and Rasmussen 2021), an open access resource of the international community of ornithologists. According to the list, there are three distinct species: the great tit ( *P. major* ), the Japanese tit ( *P. minor* ) and the cinereous tit ( *P. cinereus* ), distributed in Europe and central Asia, eastern Asia, and southern Asia and North Africa, respectively. Recent phylogeographic and demographic analyses suggest that they may have originated in the eastern Himalayas or southern Asia and subsequently dispersed to the whole of Eurasia. Among the three species, the cinereous tit is presumed to have the longest co‐existing history with local haemosporidian parasites, followed by the Japanese tit which subsequently dispersed to eastern Asia. The great tit appears to have the most recent dispersal and thus shortest evolutionary history associated with local parasites (Song et al. 2020). During the dispersal, hosts may encounter multiple parasites and establish novel host–parasite associations, subsequent within‐host speciation may occur, leading to a group of closely related lineages presenting similar prevalence in the same host. The longer parasites and their hosts coexist, the more likely it is for such speciation to occur. In this study, we mainly focus on the great tit and the Japanese tit, as no avian haemosporidian lineages have been recorded in *P. cinereus* in the MalAvi database, despite many community‐level studies including *P. cinereus* and various lineages recorded in sympatric bird species (Beadell et al. 2006; Ishtiaq et al. 2007; Gupta et al. 2019).

Here we aimed to document the phylogeographical pattern of avian haemosporidians infecting these host species and understand how the current pattern was established in relation to host population dynamics (including dispersal and subsequent speciation), based on a combination of novel field data that we collected and data from the MalAvi database. For all lineages detected in the great tit species complex, we tested their phylogenetic relationships and combined that information with data concerning their geographic and host ranges, in order to address the following questions: (1) Does the phylogeographical pattern of haemosporidian lineages correspond with the phylogeny and/or distribution of the focal host species? (2) Within each host species, do closely related lineages present more similar prevalence than expected by chance? And, (3) what were the relative roles of co‐speciation, sorting, and host range expansion in establishing these host–parasite associations?

## 2 Material and Methods

### 2.1 Sample Collection

During 2014 and 2017, wild Japanese tits ( *P. minor* ) were captured using mist nets in multiple sites within its distribution range (Table S1). Blood samples were collected from the branchial vein (never exceeding 1% of the individual's body weight) and stored in 75% ethanol until DNA extractions, which were conducted using TIANamp genomic DNA extraction kits (Tiangen Biotech Ltd., China) following the manufacture's protocol, and the quality and quantity were assessed using a Qubit 3.0 Fluorometer (Invitrogen, US) with dsDNA BR Assay. Haemosporidian infections were identified using a nested PCR protocol (Hellgren, Waldenström, and Bensch 2004) which amplified the barcoding segment of the mitochondrial *cyt b* of the parasites. The first PCR was performed using primer pair HAEMNFI (CATATATTAAGAGAAITATGGAG)—HAEMNR3 (ATAGAAAGATAAGAAATAC), and second using HAEMF (ATGGTGCTTTCGATATATGCATG)—HAEMR2 (GCATTATCTGGATGTGATAATGGT) for *Haemoproteus*/*Plasmodium* (479 bp), and HAEMFL (ATGGTGTTTTAGATACTTACATT)—HAEMR2L (CATTATCTGGATGAGATAATG) for *Leucocytozoon* (480 bp). Positive samples, determined by the existence of bands at the target length on a 2% agarose gel, were sequenced from both ends on a 3730XL automated sequencer (Applied Biosystems, USA) and assembled in Geneious Prime v. 2021.1.1 (http://www.geneious.com/). All samples were sequenced at least twice to check for possible mixed infections or false positives.

Obtained sequences were compared with those compiled in MalAvi database for taxonomic identification. Parasite haplotypes with at least one base‐pair difference in the barcoding sequence with recoded lineages were defined as novel lineages.

### 2.2 Phylogenetic Reconstruction

All haemosporidian lineages previously recorded in the great tit species complex and contained in the MalAvi database (v.2.4.1, accessed in September 2021) were selected for a global infection pattern analysis. The *cyt b* barcode sequences of the selected lineages were extracted from the ‘Fasta’ module in MalAvi (‘All sequences’) and aligned using Geneious Prime v. 2021.1.1 (http://www.geneious.com/) together with sequences obtained in this study (Table S2). For all lineages detected in host species belonging to the great tit complex, we accessed the geographical distributions and recognized host ranges in MalAvi and our field study, as well as number of previous studies (in terms of references compiled in MalAvi database) by downloading the “Host and Site table” directly into our R working environment using the malaviR package (Ellis, Bensch, and Canbäck 2017).

A Bayesian tree based on the aligned sequences was constructed using the MrBayes v.3.2.6 (Ronquist and Huelsenbeck 2003) module implemented in Geneious Prime v. 2021.1.1 (http://www.geneious.com/), with the GTR + I + G nucleotide substitution model according to the AICc model selection assessed by jmodelTest v.2.1.7 (Darriba et al. 2012). Four heated Markov chains were run simultaneously for 1 million generations and sampled every 200 generations. The first 10% of the trees were discard as “burn‐in” from the posterior distribution. The convergence of runs was checked using Tracer v. 1.4 (Rambaut and Drummond 2010) by confirming the ESS values for likelihoods and the majority of parameters was > 200. The consensus Bayesian tree was plotted in FigTree v1.4.3 (http://tree.bio.ed.ac.uk/software/figtree/, Figure S1).

### 2.3 Data Analysis

To check whether the host–parasite associations (presence/absence of a parasite in a given host) are restricted by the geographical distributions of parasites, we compared the recorded distributions of all selected lineages. Geographical overlap without infection (i.e., when a lineage was detected in host species other than the great tit species in a location where any of them could be present based on their sampling maps) would suggest that compatibility may be the main barrier of host–parasite associations, assuming that vectors are host generalists (Medeiros, Hamer, and Ricklefs 2013). To estimate the effect of sampling bias on lineage richness, we predicted the number of lineages detected at each site with increasing sampling effect using the rarefaction method in R vegan package (Oksanen et al. 2018), first with all lineages included, and then only lineages recorded at least twice. In parallel, we tested the correlation between the average prevalence (i.e., the proportion of infected individuals in all recorded samples that included sample sizes in the MalAvi database and field samples from this study) of each lineage in each host species and the number of studies in which the lineage was detected, using a Pearson's correlation test with the ‘cor.test’ function in R stats package (R Core Team 2019). For lineages that are geographically overlapped with more than one of the species (e.g., a lineage which was detected in distribution ranges of both *P. major* and *P. minor* , yet it was found exclusively in one of these two species), we compared the recorded number of studies (i.e., number of studies that recorded infections in any host) in distribution region of each species using *t* tests in the R stats package to test whether poor sampling led to not finding the lineage in a given species.

In order to investigate the role of co‐speciation, host‐range expansion and sorting events in the establishment of these host–parasite associations, we carried out a set of ancestral state reconstruction analyses under an MCMC framework using BayesTraits (http://www.evolution.rdg.ac.uk), based on the phylogenetic tree of the haemosporidian lineages (described above) and host species ( *P. major* or *P. minor* ) of the lineages. All lineages were included in this analysis, and host species was treated as discrete trait. Firstly, to estimate whether the host–parasite associations were formed randomly, we tested the transition probabilities among the two host species (i.e., a parasite infecting *P. major* dispersed to infect *P. minor* or the reverse) using Bayes Multistate model test (Pagel, Meade, and Barker 2004), firstly for all lineages and then for the lineages within each parasite genus separately. The marginal likelihood acquired from two models were compared to select for the better‐fitted one: the first did not control transition probability among different host species, while the second forced them to be equal. Log Bayes Factor (log BF) was acquired following the formula log BF = 2 × (log marginal likelihood of complex model–log marginal likelihood of simple model). A log BF less than two would support the simple model with equal transition probabilities among different hosts, otherwise, the model with different transition probabilities is preferred. A log BF exceeding ten provides strong support for the complex model. Equal transition probabilities indicate that the host–parasite associations were established without any fixed direction, while the alternative model would suggest non‐random pattern which may be the result of selection or co‐speciation. The value of transition probabilities between different hosts were calculated using the ‘fitMK’ function in phytools R package (Revell 2012) in parallel, and the history of host–parasite associations was visualized based on the best‐fit model using the ‘simmap’ function in the same R package.

We then tested the correlation between infection status in the different hosts of the great tit complex (e.g., whether a lineage can infect *P. major* is correlated with its ability to infect *P. minor* ), using the Discrete model test (Pagel and Meade 2006). In this analysis, infection status in each host species were treated as a binary trait (infection was defined as 1 and non‐infection as 0). Similar to the Multistate analysis, a log BF is obtained by comparing the likelihoods of two models: independent models assuming that infection status in the different hosts evolve independently, and dependent model which assumes the evolution is correlated. The evidence for correlated evolution can be identified by large log BF. Positive correlations would support the hypothesis that parasites kept the ability to infect each of the hosts after they split, while negative correlations would suggest that lineages are restricted to particular host species. In case that the result may be driven by rare lineages (lineages only detected in one individual), all ancestral analyses were repeated with rare lineages excluded.

To test whether closely related haemosporidian lineages present similar abilities to infect a given host, the phylogenetic signal in prevalence of lineages infecting each of the three host species separately was calculated using Pagel's λ (Pagel 1999) in the phytools R package (Revell 2012). The prevalence of a lineage was defined as the proportion of infected individuals in all collected samples, and calculated as total number of infected individuals divided by total sample size in all studies in which the lineage was recorded. As small sample size could lead to inaccurate estimation of prevalence, case studies with less than five tested individuals were excluded in this analysis. A Pagel's λ = 1 is consistent with a Brownian motion model of trait evolution, indicating that closely related lineages present more similar prevalence with each other than the prevalence of lineages chosen randomly from the phylogeny, while zero indicates that prevalence in closely related lineages is no more similar than those chosen randomly from the phylogeny (Huang et al. 2018).

Finally, we tested the hypothesis that lineages detected in *P. major* are more likely to be multi‐host parasites (i.e., have been detected in at least two host species) compared with lineages detected in *P. minor* . This may be the case if generalists were more likely to have expanded their host ranges than strict specialists (i.e., only detected in one host species) when encountered *P. major* . We compared the number of multi‐host and single‐host lineages detected in the two species using the Pearson's chi‐squared tests in the R package stats.

## 3 Results

### 3.1 Diversity of Avian Haemosporidian Lineages in the Great Tit Species Complex

A total of 304 *P. minor* samples were collected in this study, including 113 infected individuals. Taking our field data and MalAvi data together, 2762 infection cases were recorded in the great tit species complex from 72 distinct sampling sites. In all, 113 lineages were detected (59 were only recorded once), including 20 *Plasmodium*, 23 *Haemoproteus*, and 70 *Leucocytozoon* lineages (Table S2). Of these lineages, 66 have been recorded in *P. major* and 49 in *P. minor* . Two *Plasmodium* lineages, SGS1 and SYAT05, were recorded in both *P. major* and *P. minor* . Although both appear to be widely‐distributed host generalists (Hellgren et al. 2015), the former was frequently recorded in both host species, while the latter was only recorded once in each host species.

The recorded number of infected individuals in *P. major* was much larger than *P. minor* (Table S2), but the difference was mainly driven by a few studies in which hundreds of samples were tested (Wood et al. 2007; van Rooyen et al. 2013; Dubiec et al. 2016; Schumm et al. 2019; Ellis et al. 2020; Lynton‐Jenkins et al. 2020). Rarefaction analysis show that the diversity of lineages may be underestimated, but when rare lineages (lineages only detected once, 31 for *P. major* and 29 for *P. minor* ) were excluded, estimated lineage diversity stabilized before reaching the actual sample size, suggesting that our data can represent the host–parasite associations in this system, even if one or two lineages may remain undetected (Figure S2). For both *P. major* and *P. minor* , the mean prevalence (i.e., prevalence combining data in MalAvi and in the current study) of each lineage was not correlated with the number of studies identifying the lineage ( *P. major* : *r* = 0.15, *t*_64_ = 1.22, *p* = 0.23; *P. minor* : *r* = −0.04, *t*_46_ = −0.28, *p* = 0.77), indicating that sampling bias has little effect on the results.

Among all lineages detected in *P. major* , ten have been recorded in a geographical region where *P. minor* individuals were sampled (according to study sites recorded in MalAvi database) and eight of those have not been recorded in the latter. For these lineages, there were more records (according to MalAvi) in regions where *P. major* was distributed compared to the regions where *P. minor* (*t*_7_ = 2.79, *p* = 0.01) was distributed. Thus, the lack of infection was either due to poor parasite sampling or alternatively, the lineages may simply be rare in the areas where *P. minor* is present and *P. major* is absent. Similarly, 14 lineages detected in *P. minor* were found within the ranges where *P. major* was sampled and tested, of which 12 were absent in *P. major* , but showed no significant difference in recorded number between regions where the two host species were distributed (*t*_11_ = 0.97, *p* = 0.35). Besides, seven lineages have been recorded in geographical distribution of *P. cinereus* , but without definite sampling records we do not know whether the absence of infection results from a lack of sampling.

Host ranges differed significantly among the 113 detected lineages. Almost half of them (52 lineages) have only been detected in one host species and therefore are likely to be specialists, while 20 presumed generalist lineages have been detected in at least ten host species. Among them, 14 lineages have been detected in other *Parus* species, 11 in *Parus palustris* and 5 in *Parus venustulus*. Lineages detected in *P. minor* but not in *P. major* seem to present slightly narrower identified host ranges (in terms of smaller number of recorded host species, mean ± SE = 5.8 ± 1.9) than those detected only in *P. major* (7.2 ± 1.6) but the difference is not statistically significant (Wilcoxon's test, *p* = 0.56). When separating the lineages by parasite genus, *Haemoproteus* and *Leucocytozoon* lineages restricted to *P. major* tended to infect more host species (*Haemoproteus*: 10.8 ± 3.8; *Leucocytozoon*: 3.9 ± 1.1) than those only detected in *P. minor* (*Haemoproteus*: 4.2 ± 1.1; *Leucocytozoon*: 2.2 ± 0.5, Wilcoxon's test, *Haemoproteus*: *p* = 0.19; *Leucocytozoon*: *p* = 0.08) although the former is not significant due to multiple specialist parasites restricted to *P. major* , while no difference was detected in *Plasmodium* lineages ( *P. major* : 17.2 ± 6.9, *P. minor* : 18.6 ± 10.1, *p* = 0.79). Meanwhile, nearly half the lineages (31 out of 66) detected in *P. major* were also recorded in *Cyanistes caeruleus* , another well‐studied tit with a distribution range largely overlapped with *P. major* .

### 3.2 Evolutionary Pattern

On the phylogenetic tree, lineages detected in different locations and different hosts often clustered together but there are many instances of host range expansion and long‐distance dispersal (Figure S3).

**FIGURE 2:**
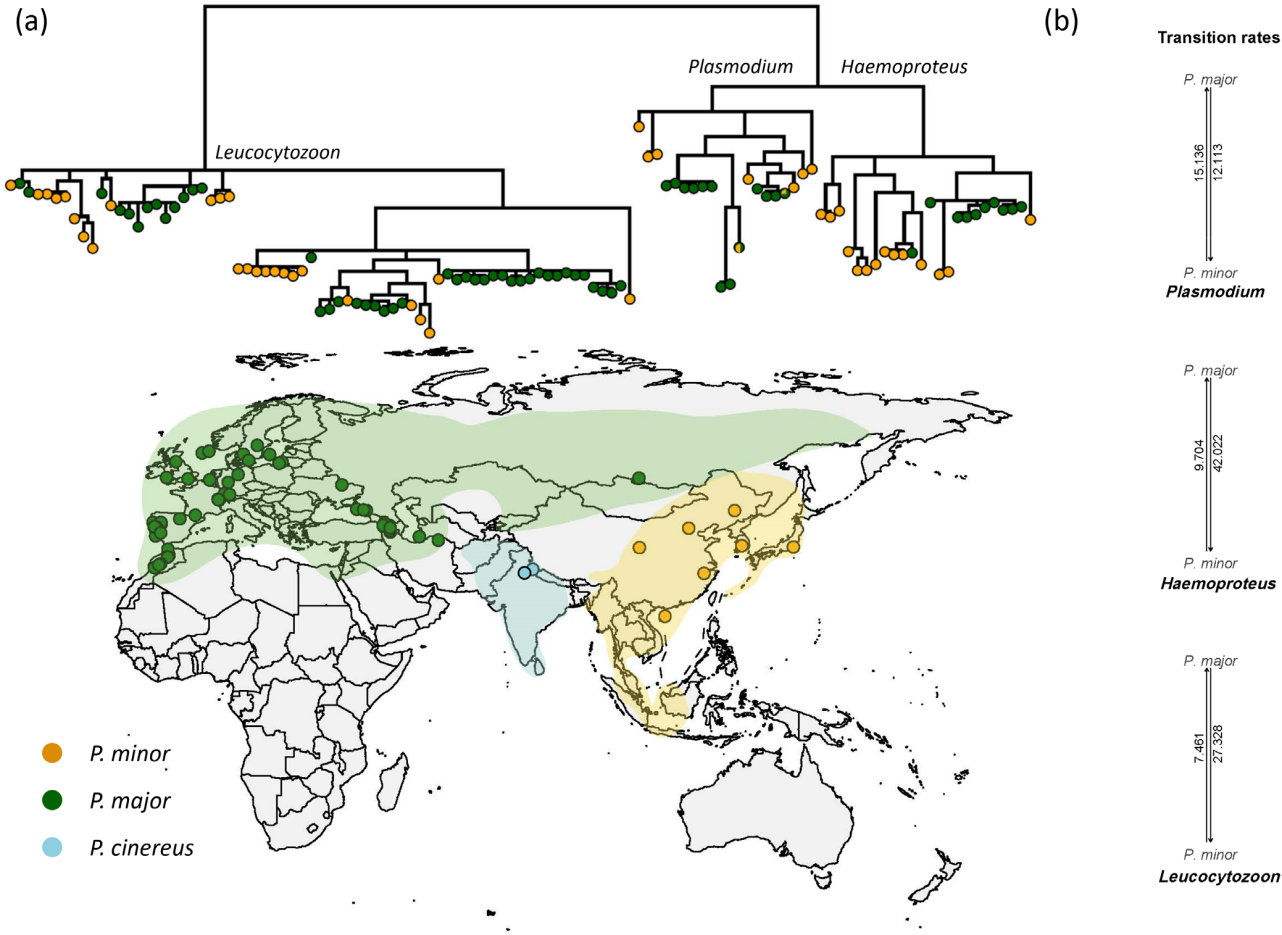
Phylogeographic pattern of all haemosporidian lineages recorded in host species belonging to the great tit species complex ( *P. major* , *P. minor* and *P. cinereus* ). (a) Geographical distribution (colored area) and sampling sites (points) of the three host species, including those recorded in MalAvi database and this study, colors represent host species within the great tit complex. *P. cinereus* individuals have been sampled in previous studies without infection record. (b) Transition probabilities among different host species in each parasite genera.

Ancestral reconstruction analysis by BayesTraits did not detect any significant difference in transition probability among host species. The log marginal likelihood of the two models differed by 0.7, a log BF lower than two suggesting that the simple model was preferred, that is the probability at which lineages infecting *P. major* dispersed to *P. minor* is equal to the reverse. When the three genera of parasites were treated separately, the same result was obtained for *Plasmodium* (log BF = 1.5) and *Haemoproteus* (log BF = 0.34), while for *Leucocytozoon* a weak significance was detected (log BF = 3.2). This result was supported by the transition rates calculated by ‘fitMK’ analysis in phytools package, which detected a higher transition probability from *P. major* to *P. minor* than the reverse for *Leucocytozoon* lineages, and similar transition probabilities for *Plasmodium*. However, for *Haemoproteus* lineages, the transition probabilities between species obtained from fitMK analysis differed greatly (Figure 2b). The patterns changed slightly when rare lineages were excluded, for both *Plasmodium* and *Haemoproteus*, the transition probability from *P. minor* to *P. major* increased a lot, from 14.284 to 28.038, and from 8.134 to 38.957, respectively (Figures 2,S3).

On the other hand, discrete analysis detected a significant negative correlation between infection status of the two hosts (log BF = 158.5, which is much larger than 10 and therefore provides strong support for the complex model), suggesting that haemosporidian lineages are likely to be restricted to one species, a finding that we also see in the raw data with only two lineages infecting both species.

Prevalence was calculated for each lineage (two were excluded due to small sample size, both belong to *Plasmodium*) to test for phylogenetic signal (Table 1). For lineages detected in *P. minor* , we detected a strong phylogenetic signal (i.e., closely related lineages presented similar prevalence). When lineages belonging to different genera were treated separately, a similar pattern was observed in *Plasmodium* and *Haemoproteus* lineages (although not significantly different from zero in *Haemoproteus*), while in *Leucocytozoon* the phylogenetic signal was close to zero. When zero prevalences (lineages not detected in focal host) were excluded, the phylogenetic signal of *Haemoproteus* dropped rapidly (from 0.61 to 0.08), but that for *Plasmodium* did not change. For *P. major* , no significant phylogenetic signals were detected, either for all lineages or within either genus (Table 1).

**TABLE 1:** Phylogenetic signal of the haemosporidian prevalence in *Parus major* and *Parus minor* host species for each haemosporidian genus separately.

	*Parus major*			*Parus minor*		
	Lineage no.	λ	*p*	Lineage no.	λ	*p*
*Plasmodium*	13	< 0.01/< 0.01	1/1	9	**1.00**/**1.00**	**< 0.01**/**0.01**
*Haemoproteus*	10	< 0.01/< 0.01	1/1	14	**0.61**/0.08	0.02/0.86
*Leucocytozoon*	43	< 0.01/< 0.01	1/1	26	< 0.01/< 0.01	1/1
Total	66	0.03/0.06	0.29/0.18	49	**0.97**/< 0.01	**< 0.01**/1

*Note:* Haemosporidian lineages with a sample size of less than five were excluded in this analysis to potentially control for erroneous results caused by sampling bias. In case that result may be driven by lineages without infection (prevalence equal to zero), phylogenetic signals were calculated with these lineages included and excluded in parallel (presented as with/without zero prevalence), strong signal and significantly different from zero are marked in bold.

## 4 Discussion

In this study, we presented the global diversity and phylogenetic relationships of avian haemosporidian lineages detected in the great tit species complex (namely *P. major* and *P. minor* , while no haemosporidian lineages were recorded in *P. cinereus* ), one of the most well‐studied avian hosts. The host–parasite assemblages showed a clear geographical signal, but the phylogeny of detected haemosporidian lineages did not cluster by either host species or geographical distribution. Hence, many examples of host switches were found, and the transition probability between host species was nearly equal between *P. major* and *P. minor* . Furthermore, among the lineages that are geographically overlapping with more than one host species, the majority (22 out of 25) were restricted to either *P. major* or *P. minor* . Interestingly, there are no records of parasites infecting *P. cinereus* in the MalAvi database. This may suggest that the observed pattern in host–parasite assemblages in this species complex was mainly shaped by many instances of host‐range expansion of parasites during host dispersal, followed by local adaptation to specific hosts. If these parasites or their ancestors predate the split between *P. minor* and *P. cinereus* , it would be a striking example of sorting (i.e., missing the boat) that led to *P. cinereus* not being infected.

*Parus cinereus* is thought to have not dispersed from the regions where the species complex originated (Zhao et al. 2012; Song et al. 2020). From our data we cannot ascertain if the ancestor of the three species was infected by parasites that were subsequently lost when *P. cinereus* diverged (sorting), or if the parasites colonized *P. minor* or *P. major* after they dispersed to their current distribution and split from *P. cinereus* (host range expansion). Up till now, studies known to have tested *P. cinereus* are scarce, therefore, the lack of infection records in this species might barely due to poor sampling. However, various community‐level studies have been carried out within the distribution range of this species, in which multiple lineages were recorded, both in migratory and resident species. Infections in local resident species implies that there are competent vectors for parasite transmission, that is, compatibility was the main barrier for parasite to infect the focal species. As negative results are rarely published, there may be more *P. cinereus* samples tested previously, although not as extensive as *P. major* . There are other examples of resistant bird species that have not been found to be infected with avian haemosporidians or are rarely infected despite abundant sampling (Ellis et al. 2020). One hypothesis is that evolutionary distinctiveness of a host species is negatively related to parasite prevalence and diversity (Huang et al. 2015; Ellis et al. 2020), although this was only shown to have support for *Haemoproteus* parasites in a recent study (Ellis et al. 2020). As molecular evidences have identified the distinct role of *P. cinereus* (Song et al. 2020), it might be resistant to infection because it is relatively evolutionarily distinct from the other species in the complex. Or, more likely, *P. cinereus* had diverged significantly from its close relatives in immune system during the long‐term evolution, making it highly resistant to haemosporidian infections. Further studies on immune‐related genes (e.g., MHC genes, etc.) are required to test this assumption.

The assemblages of parasites differed between the two focal species. Over 100 haemosporidian lineages were detected in the great tit species complex, nearly one‐fifth of them can be defined as generalist parasites (according to no less than 10 recorded host species in the MalAvi database). However, only two lineages were detected in both *P. major* and *P. minor* , while all the others were restricted to one of them. As haemosporidian parasites are vector‐transmitted, the establishment of host–parasites associations may be largely affected by vector dynamics. What's more, dietarian vectors are the only hosts where sexually reproduction occurs (Santiago‐Alarcon, Palinauskas, and Schaefer 2012), difference in vector composition may lead to parasite speciation. Additionally, climatic differences in different geographical locations can influence the reproduction and development of vectors, and further affect parasite transmission. However, up till now we still know little on the diversity of vectors in most study sites, not to say their preferences in biting bird hosts. We call for more vector‐focused studies to enhance our understanding of the factors driving the formation of current host–parasite associations.

For lineages that were only detected in *P. major* , most (87%) were not recorded in regions where *P. minor* or *P. cinereus* is distributed, and a similar pattern is seen for lineages restricted to *P. minor* (74% only recorded in regions where *P. minor* is distributed). This suggests that dispersal limitation of parasites may play a role in determining the current host–parasite associations, as addressed in previous studies (Soares, Latta, and Ricklefs 2017). Interestingly, a number of geographically restrict lineages have been detected in other sympatric *Parus* species. If these lineages present similar abilities when infecting close related hosts as presumed in previous studies (Clark 2018; Huang et al. 2018), they may be able to infect all three species in the great tit species complex if they were to come into contact. Infection experiments could be carried out in future to test this assumption. Moreover, sympatric *C. caeruleus* harbored nearly half the lineages detected in *P. major* , addressing the role of geographical distribution in establishing host–parasite associations. However, more than 20 lineages are geographically overlapping with at least two host species but only infect one of them. For some lineages, the lack of infection might due to poor parasite sampling or heterogeneous distribution in regions occupied by the three host species. Another cause should be low compatibility between host and parasite (Combes 2001) and is indicative of localized adaptations, plausibly of both hosts and parasites, indicating that local adaptation to hosts may have stronger effect in shaping host–parasite associations than dispersal limitation of parasites.

By visual inspection, haemosporidian lineages detected in the same host clustered together on the phylogenetic tree (Figure 2). Rarefaction analysis shows that increased sampling size may lead to higher number of detected lineages but only rare ones, therefore the observed pattern of host–parasite assemblages would be close to the natural pattern. Generally, no clear host‐related patterns were detected in the phylogeny of *Plasmodium* lineages, which is also suggested by the ancestral reconstruction analysis that found the transition probabilities to be almost equal between the different host species. Thus, the formation of host–parasite associations does not seem to have tracked the evolution of host species (i.e., co‐speciation) in the *Plasmodium*—great tit system. Instead, it may have been shaped by a combination of random sorting events (Figure 1b) after the host speciation and multiple instances of host‐range expansion of local parasites in newly colonized region of the hosts (Figure 1c). Most of the *Leucocytozoon* lineages appear to be host specialist with only one recognized host species, and lineages detected in the same host clustered together, suggesting that within‐host speciation have occurred multiple times during the evolutionary history. As for *Haemoproteus* lineages, the transition probability from *P. major* to *P. minor* appeared to be higher than the reverse, although the simple model with equal transition probability was preferred by BayesTrait analysis. Because the Bayes ancestral analyses may not consider the difference in encounter frequency between parasites and their potential hosts, the result may be biased by the heterogeneous geographical distribution of lineages. When rare lineages were excluded, the transition probabilities between two species were close to equal. Despite the discrepancy between different analyses, both results indicate unique evolutionary patterns among three genera of haemosporidians, probably due to differences in life history (Valkiūnas 2005), host specificity (Hellgren, Pérez‐Tris, and Bensch 2009), and vector behavior (Santiago‐Alarcon, Palinauskas, and Schaefer 2012).

According to molecular evidence, *P. major* separated from the common ancestor and subsequently dispersed to new geographical regions during the speciation process (Zhao et al. 2012). Therefore, lineages only detected in *P. major* are likely to be the result of host‐range expansion of parasites, that is, host–parasite associations were established when parasites in current geographical regions were compatible with *P. major* . Lineages that were restricted to *P. major* are more likely to be multi‐host parasites (due to the lack of infection intensity data and biased sample size of host species, we roughly defined host specificity of a given parasite by number of host species it can infect) while more single‐host parasites were detected in *P. minor* , indicating high potentials of host range expansion in *P. major* , apart from the bias of sampling effect. Among lineages detected in *P. major* , 11 were geographically overlapping with *P. minor* and/or *P. cinereus* without infecting either of them. However, these lineages had relatively few records in regions where these two species were distributed, suggesting that low encounter frequency might be a barrier to establishing host–parasite associations. In multi‐host communities, generalist parasite lineages are often more abundant than others, and therefore more likely to expand to newly encountered hosts (Carlson et al. 2022), including migrants and recently encountered immigrants (Huang et al. 2022), such as *P. major* .

While for lineages that were only detected in *P. minor* but not in *P. major* , there are two possible explanations. The first is sorting events, that is, lineages harbored by the common ancestor “missed the boat” during the dispersal of *P. major* , or arrived to the new regions but could not transmit and therefore got extinct. Alternatively, the lineages may have colonized *P. minor* and establish novel associations after the dispersal of *P. major* and therefore have no chance to encounter the latter. For lineages that were geographically overlapping with both *P. major* and *P. minor* , the record frequency (number of studies in which the lineages were recorded) was similar in both regions, suggesting that they may have equal opportunities to encounter both hosts. However, 85% of them were only detected in *P. minor* , supporting the assumption that those host–parasite associations are formed after the host divergence. It is worth noting that when rare lineages (lineages recorded only once in these species) were excluded, the transition probability from *P. minor* to *P. major* increased rapidly but not the reverse. Comparing with relatively common lineages, rare lineages in *P. minor* present lower prevalence, suggesting that they may have encountered their host more recently and not yet well adapted. This evidence is consistent with the assumption that lineages detected only in *P. minor* established the host–parasite associations after the dispersal of *P. major* . On the other hand, common lineages detected in *P. minor* maybe more ancient than those in *P. major* (Figure S3), suggesting that sorting can be the main reason for their absence in *P. major* .

When prevalence was taken into consideration, lineages detected in *P. minor* presented much stronger phylogenetic signal than those in *P. major* , that is, closely related parasites present similar abilities in infecting the *P. minor* , which might be the result of with‐in host diversification of parasites. Meanwhile, compatibility between hosts and parasites are thought to increase during long‐term local co‐evolution (Samuel et al. 2015), and enhance the phylogenetic‐related infection patterns. Therefore, *P. minor* might have had a longer co‐evolutionary history with its parasites, corresponding to previous finding that the dispersal of *P. minor* occurred earlier than that of *P. major* . Unfortunately, we are unable to know the lineages that infected the common ancestor due to a lack of an agreed upon haemosporidian molecular clock to time lineage divergence (Bensch et al. 2013). Besides, the strong phylogenetic signal for *Haemoproteus* lineages were likely driven by lineages without infection record, once those lineages were removed, the signal dropped to approach zero. Moreover, prevalence of a certain lineage in focal hosts might be related with its host specificity, as specialist parasites are likely to be better adapted to their hosts than generalist parasites do, and generalist parasites often present significantly higher prevalence in main hosts than in sporadic hosts. Despite of these, our result suggest that sorting and subsequent host‐range expansion are the main drivers during specification of the great tit species complex, and provide a new insight in understanding the host–parasite co‐evolutionary history.

## 5 Conclusion

In this study, we investigated the establishment of host–parasite associations in relation to the population dynamic of host species in a well‐studied avian species complex. The observed pattern differs slightly among parasite genera. But generally, it appears that sorting events and host‐range expansion of parasites have played the main role in establishing host–parasites associations during host divergence and dispersal, rather than co‐speciation. We have addressed the importance of host population dynamic and phylogeography in the establishment of host–parasite associations, and call for more host‐centric studies in investigating host–parasite associations.

## Author Contributions

**Xi Huang:** conceptualization (lead), data curation (equal), formal analysis (lead), funding acquisition (equal), investigation (lead), methodology (lead), project administration (lead), writing – original draft (lead). **Vincenzo A. Ellis:** conceptualization (equal), data curation (equal), formal analysis (supporting), methodology (supporting), writing – review and editing (equal). **Yangyang Peng:** data curation (equal), investigation (equal), resources (equal). **Farah Ishtiaq:** data curation (equal), resources (equal), writing – review and editing (supporting). **Haitao Wang:** resources (equal), writing – review and editing (supporting). **Wei Liang:** resources (equal), writing – review and editing (supporting). **Qiang Wu:** resources (equal), writing – review and editing (supporting). **Staffan Bensch:** resources (equal), validation (equal), writing – review and editing (supporting). **Lu Dong:** conceptualization (equal), funding acquisition (equal), methodology (equal), writing – review and editing (equal).

## Conflicts of Interest

The authors declare no conflicts of interest.

## Supporting information


**Table S1.** Summary of samples collected and tested for haemosporidian infections in this study.

**Table S2.** Host range and geographical distribution of lineages detected in the *Parus major* species complex. Infection no., total number of infected individuals; Record no., number of references compiled in the MalAvi database.

**Figure S1.** Phylogenetic tree with posterior probabilities of all haemosporidian lineages recorded in the great tit species complex, novel lineages detected in this study are marked in blue.

**Figure S2.** The predicted number of lineages detected in each site with increasing sampling effect using the rarefaction method, (a) in *P. major* with all lineages included, (b) in *P. minor* with all lineages included, (c) in *P. major* only include lineages recorded at least twice, (d) in *P. minor* only include lineages recorded at least twice.

**Figure S3.** Phylogeographic pattern of haemosporidian lineages recorded in at least two host individuals belonging to the great tit species complex and transition probabilities among different host species in each parasite genera.

## Data Availability

Barcoding sequences of novel lineages have been uploaded to GenBank. Original data and R code are available at Dryad: https://doi.org/10.5061/dryad.3j9kd51tg, https://datadryad.org/stash/share/bu1ehCCn9PO1m‐4vmYc8nCi_‐jYITjnYckT7Q0afbEM.
