# Supplementary figures and images for: The Role of Host‐Range Expansion and Co‐Speciation in Host–Parasite Associations With the Divergence of the Great Tit Species Complex
Source: Ecol Evol. 2025 Jan 21;15(1):e70859. doi: 10.1002/ece3.70859 (PMC11747346; doi:10.1002/ece3.70859)

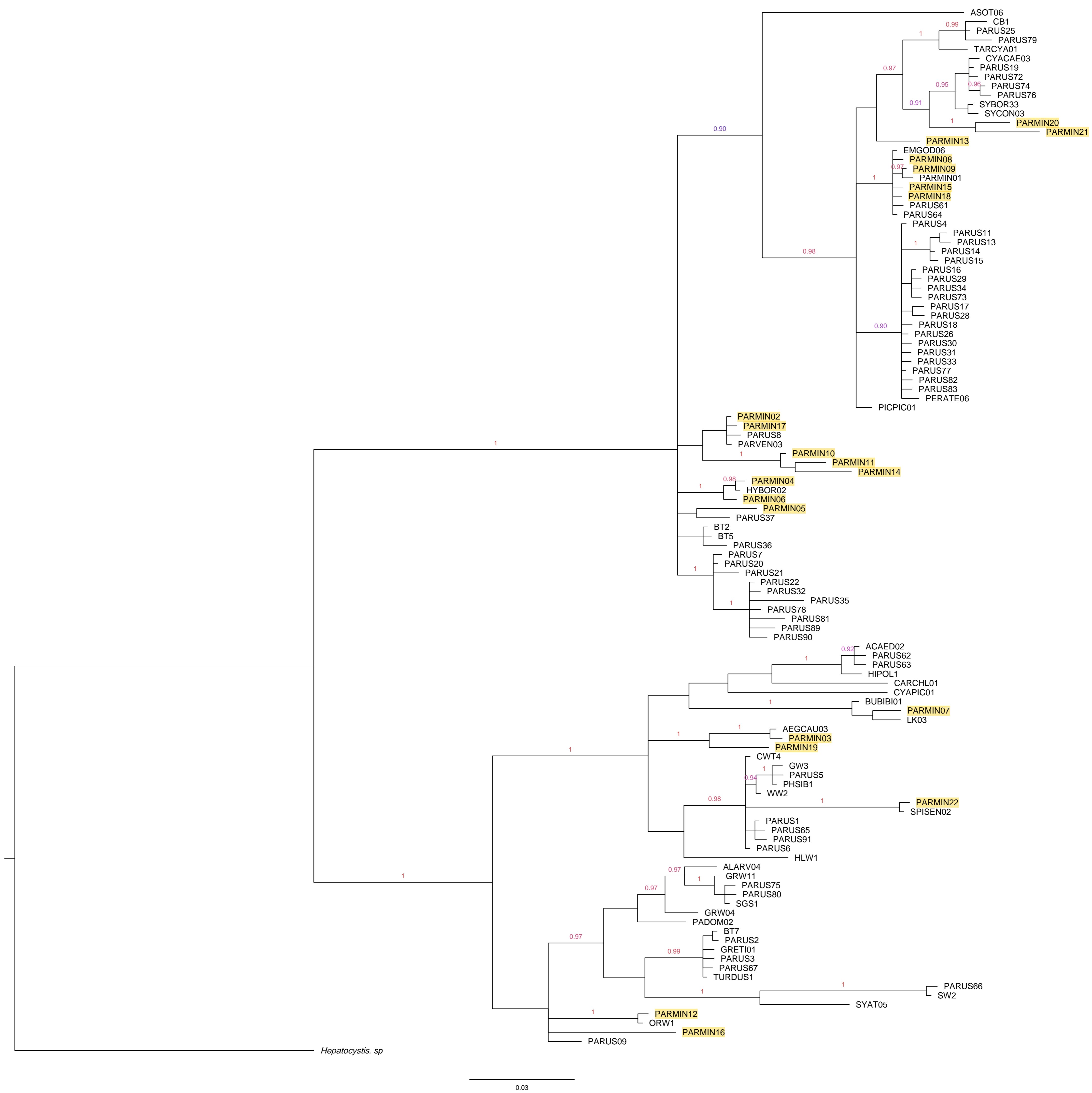

Supplement: Supplementary file 3 — Figure S1. Phylogenetic tree with posterior probabilities of all haemosporidian lineages recorded in the great tit species complex, novel lineages detected in this study are marked in blue. [file ECE3-15-e70859-s004.pdf]

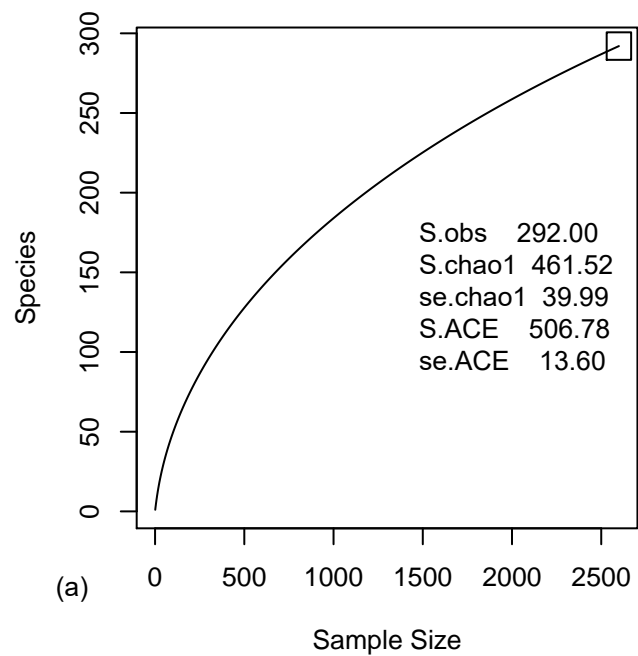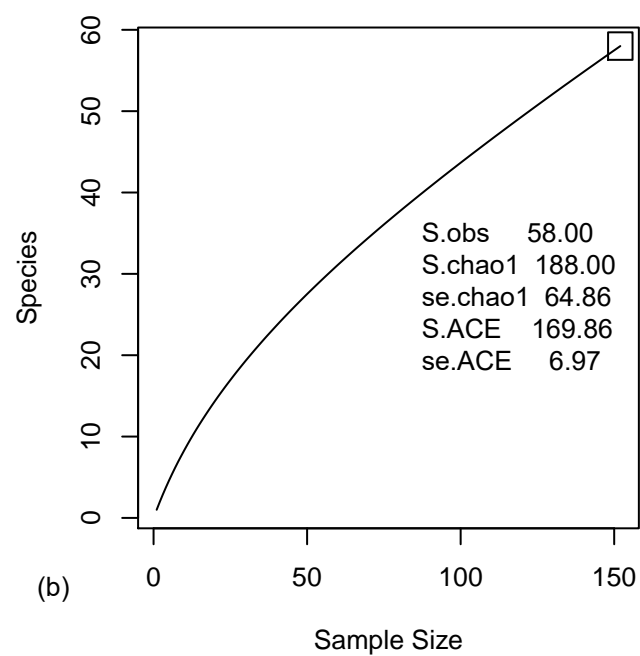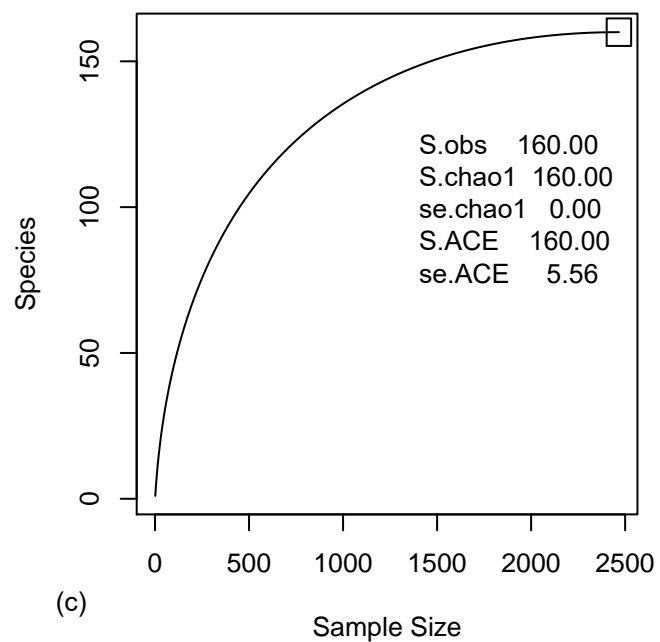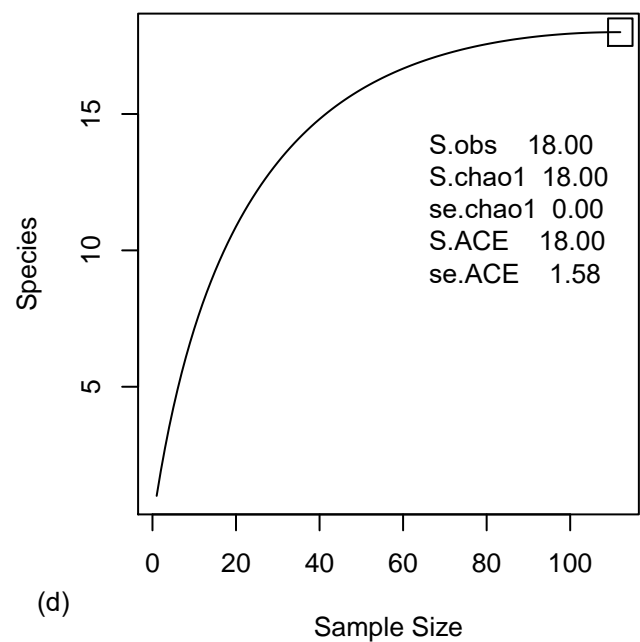

Supplement: Supplementary file 4 — Figure S2. The predicted number of lineages detected in each site with increasing sampling effect using the rarefaction method, (a) in P. major with all lineages included, (b) in P. minor with all lineages included, (c) in P. major only include lineages recorded at least twice, (d) in P. minor only include lineages recorded at least twice. [file ECE3-15-e70859-s001.pdf]

(a)

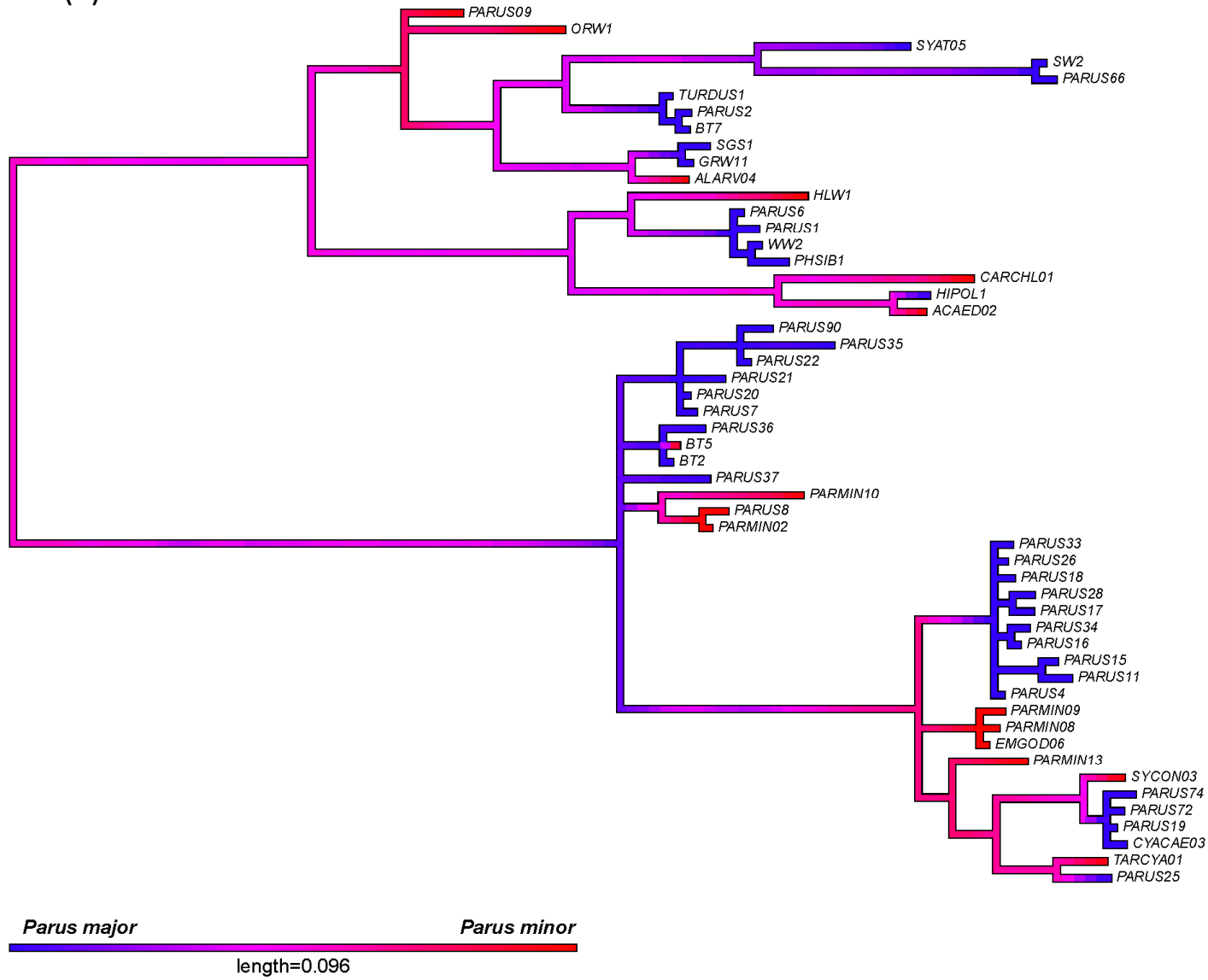

(b)

Transition rates

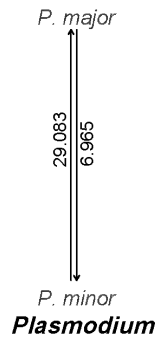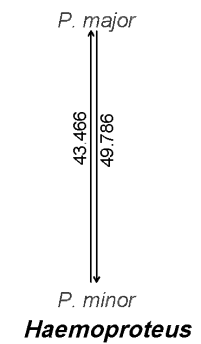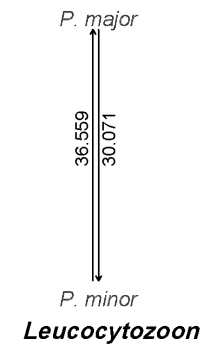

Supplement: Supplementary file 5 — Figure S3. Phylogeographic pattern of haemosporidian lineages recorded in at least two host individuals belonging to the great tit species complex and transition probabilities among different host species in each parasite genera. [file ECE3-15-e70859-s002.pdf]
